# Stiffening the human foot with a biomimetic exotendon

**DOI:** 10.1038/s41598-021-02059-8

**Published:** 2021-11-23

**Authors:** Ryan C. Riddick, Dominic J. Farris, Nicholas A. T. Brown, Luke A. Kelly

**Affiliations:** 1 Centre for Sensorimotor Performance, University of Queensland, Brisbane, QLD 4072 Australia; 2 Sport and Health Sciences, University of Exeter, Exeter, EX4 4PY UK; 3 Faculty of Health, University of Canberra, Canberra, ACT 2617 Australia

**Keywords:** Biomechanics, Systems biology, Biomedical engineering

## Abstract

Shoes are generally designed protect the feet against repetitive collisions with the ground, often using thick viscoelastic midsoles to add in-series compliance under the human. Recent footwear design developments have shown that this approach may also produce metabolic energy savings. Here we test an alternative approach to modify the foot–ground interface by adding additional stiffness in parallel to the plantar aponeurosis, targeting the windlass mechanism. Stiffening the windlass mechanism by about 9% led to decreases in peak activation of the ankle plantarflexors soleus (~ 5%, p < 0.001) and medial gastrocnemius (~ 4%, p < 0.001), as well as a ~ 6% decrease in positive ankle work (p < 0.001) during fixed-frequency bilateral hopping (2.33 Hz). These results suggest that stiffening the foot may reduce cost in dynamic tasks primarily by reducing the effort required to plantarflex the ankle, since peak activation of the intrinsic foot muscle abductor hallucis was unchanged (p = 0.31). Because the novel exotendon design does not operate via the compression or bending of a bulky midsole, the device is light (55 g) and its profile is low enough that it can be worn within an existing shoe.

## Introduction

While the ankle, knee, and hip joints are often the focus of exoskeleton research, perhaps the most broadly used wearable assistive device throughout history has been the shoe, with some of the earliest footwear thought to be woven sandals worn over 9000 years ago^1^. The foot is considered an important contributor to the energetics of gait through both the dynamics of its joints and through energy losses from soft tissue vibrations^2–7^. Shoes have historically been designed for fashion, comfort, and protection. Only recently have they been successfully designed to increase performance in dynamic activities such as running beyond what the bare foot is capable of^8–11^. These shoes generally stiffen the foot in some way, but since they are composite devices which may affect the dynamics of several parts of the foot simultaneously, the exact mechanism by which they increase performance is not clear.

Referencing the spring-like mechanics observed in running, shoes designed for performance often seek to incorporate elasticity into the shoe^9,12^. Because shoes generally have minimal capacity to constrain the movement of the foot inside the shoes, one performance enhancing strategy is to tune the compressive elasticity of a shoe midsole, which can lead to improvements in running economy^10,11^. Another approach is to incorporate stiff carbon fiber plates into a shoe to increase the longitudinal bending stiffness of shoes^13^. This strategy has led to increases in running economy over designs which only target compression mechanics^8,9^. These plates serve to stiffen the metatarsophalangeal (MTP) joint within the foot as the leg pushes off from the ground. A third approach is to insert insoles into shoes shaped to follow the contour of the arch; however, they are usually designed to provide comfort or support instead of targeting energy absorption and return^14,15^. For example, rigid insoles designed specifically to block the motion of the arch provide little energy return and can increase the cost of transport in running by about 6%^7^.

While embedded carbon fiber plates are thin enough to bend, thereby stiffening the MTP, they are practically rigid in regards to the longitudinal axis of the foot. When the foot is tied to such a rigid object, it restricts the elongation of the foot. This restriction of the foot presumably leads to the reduction in compression and elastic work of the arch during running when such shoes are worn^16^. To the best of our knowledge, there are currently no devices which target these two major joints synchronously to increase the overall elastic work of the foot without restricting the motion of the arch. Part of this absence is likely due to the pervasive methodology in biomechanics of interpreting joint dynamics without considering structures which span multiple joints. It has been shown that the actions of other bi-articular muscles such as gastrocnemius and rectus femoris greatly affect estimates of work performed by the joints of the leg^17–20^. When observing the MTP joint in isolation, it appears that it is dissipating energy as the leg pushes off of the ground and therefore costs the body energy^21,22^. However, the windlass mechanism couples the motions of the arch and MTP together with the elastic structure of the plantar aponeurosis^4,23^. Within this broader system, the energy “lost” at the MTP can be instead understood as energy transferred to the arch of the foot^4^. Analyzing the action of these two joints together or modelling the plantar fascia directly show that the plantar fascia operates nearly elastically when stepping on level ground^4,6,24^.

From this viewpoint, footwear that can reduce metabolic cost is likely to operate by reducing the muscle cost of producing a certain stiffness within the foot (as opposed to reducing energy dissipation). One possibility is that it could reduce activation of the muscles of the foot to produce a given level of stiffness at the MTP^25,26^. Additionally, an increase in foot stiffness could produce a longer and stiffer mechanical lever arm for proximal joints to push off of the ground^27–29^, potentially driving increases in efficiency by reducing the contraction velocity required by the ankle plantar flexors.

These ideas suggest that a device which aims to increase performance via an increase in foot stiffness would benefit from targeting the plantar fascia and intrinsic muscles directly. From here on, we refer to this grouping of tissues and muscles acting via the windlass mechanism as the plantar Muscle Tendon Unit (MTU). Basketball players have been shown to have a higher passive stiffness at the MTP when compared to other athletes^30^. This suggests that that there is an activity-dependent benefit to passively stiffening the plantar MTU, since it is the predominant structure affecting the passive dynamics of the MTP. Devices which passively stiffen muscle–tendon pathways have been shown to be effective at reducing muscle activity at the ankle, knee, hip, and lumbosacral joints across a variety of tasks^31–36^. Hopping is particularly well suited for studying such devices, since the joints of the foot and ankle joints operate nearly elastically^37^. Passive exoskeletons at the ankle have been shown to reduce activation of the plantarflexing muscles of the ankle ^38^ and metabolic cost^39^ during bilateral hopping. While hopping is a less common activity, it is also characterized by a bouncing motion^40^ and avoids introducing more complicated and non-linear joint dynamics when compared to running^38,41^. Running also contains a ballistic phase in which the leg is rapidly swung forward to reposition the foot^42^, dynamics which most assistive devices are not designed to target, and in fact may hamper due to the increased mass added to the leg from the device^43–45^.

**Figure 1:**
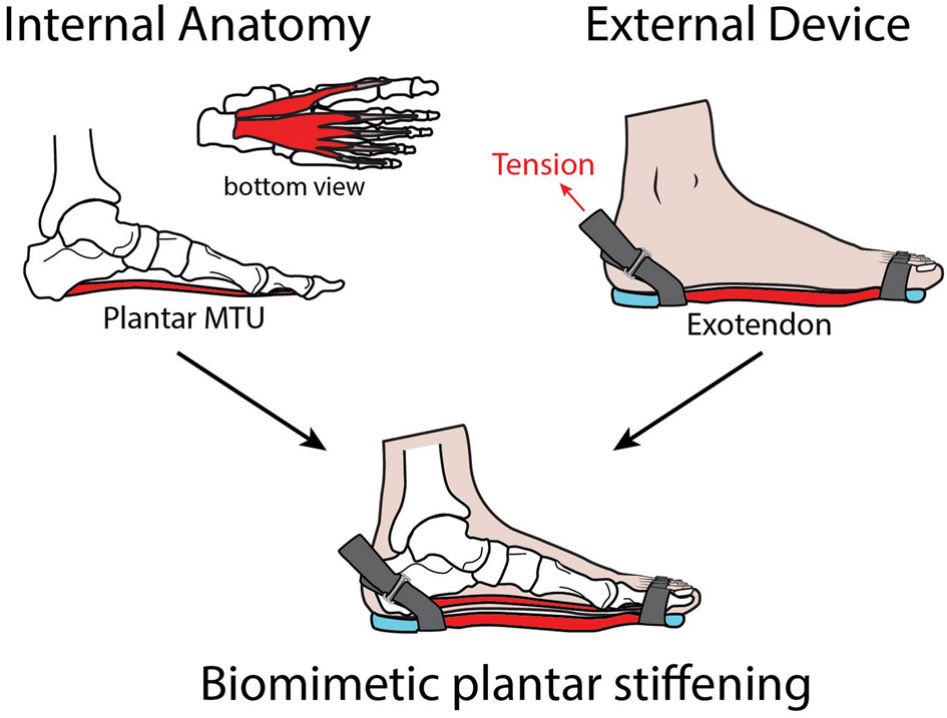
A diagram showing how the exotendon attaches to the bottom of the foot for the purpose of stiffening the plantar MTU during hopping. A strap is used to tension the exotendon such that it produces force in parallel with the plantar MTU as soon as the foot make contacts with the ground. The plantar fascia and exotendon act in tension (shown in red) during hopping and are designed to generate substantial force across the joints of the foot.

Our objective was to conduct an experiment in which we could increase plantar MTU stiffness and test whether this can reduce muscle activation of the foot and ankle during hopping. We designed a soft device with a highly elastic exotendon that mimics the anatomy of the plantar MTU instead of encapsulating it like typical footwear, or blocking the motion of the arch, like insoles. The exotendon is tensioned in parallel with the path of the plantar MTU to stiffen the foot (Fig. 1). We first verify that such a device is capable of stiffening the foot during a cyclic, bilateral hopping task. We then discuss the effects of the device on muscle activation and mechanics of the leg, and its potential benefits as augmentation for standard footwear.

## Methods

### Participants

Informed consent from 9 volunteering participants was obtained prior to the start of the experiment. Both the process of obtaining informed consent and the experimental protocol itself were carried out following the guidelines provided by the University of Queensland Human Research Ethics Committee. The experimental protocol was approved by this committee and is detailed below. Participants were to be excluded if they had a lower limb injury within the six months prior to the experiment, or a known neurological impairment. The participants had a *mean* ± *standard deviation* age of 27.2 ± 2.8 years, height of 180.8 ± 4.4 cm, and mass of 78.6 ± 11.9 kg.

### Exotendon design

A simple diagram of the device can be found in Fig. 1, with a detailed description of the device provided below. The main portion of the device was a single piece of highly resilient thermoplastic polyurethane (TPU) cut into the shape of the plantar aponeurosis, including individual slips that extend distally to each toe. This served both as the exotendon, increasing the stiffness of the foot, as well as creating an interface or barrier between the foot and the ground. Five different samples of the exotendon material with thickness of 2.56 ± 0.10 mm were stretched in tension at a rate of 120 mm/min while a load cell measured the resultant forces (Instron 5543, Norwood MA, USA). The samples were found to have an average elastic modulus of 11.56 ± 1.75 MPa.

To attach the distal end of the exotendon to the foot, 2 cm wide cloth elastic loops were sewn and glued to each of the TPU toe slips, through which the toes could be inserted. The diameters of the loop were set such that upon insertion of the toes, the elastic banding within the loops provided as high an amount of pretension as possible without inducing discomfort while wearing the device. This pretension was necessary to minimize the amount of stretching the loops endured during use, such that energy transfer between the exotendon and the human foot could be maximized. At the proximal end of the exotendon, a 3 cm wide nylon webbed strap was sewn and glued to the TPU. This strap wrapped around the back of the calcaneus. On one end of the strap was sewn a slider mechanism, through which the end of the other strap could be fed through and tightened around the foot. The tightening of this strap served to attach the device to the foot of each participant, as well as to tension the exotendon. This attachment design enabled the use of a single device to be adjusted to participants with different sizes of foot. This design constrained the Young’s modulus of the device to be constant across participants. In contrast, the overall net stiffness of the device was therefore inversely proportional to foot length (since a larger portion of the exotendon would be stretched for participants with longer feet).

A soft closed cell polyethylene foam was adhered with double-sided tape to the heel of each participant in between the device and their foot in order to reduce the tendency of the device to slip. With the participants’ foot in a resting, neutral posture, the strap was tightened until the device just began to draw the toes of the participant into flexion (such that there was no slack but also no significant pretension in the device). The strap was then locked in place at this level of tension with Velcro that was sewn into the strap. After each trial, the device was checked to ensure no slipping had occurred. The total mass of the device was 55 g.

### Protocol

Participants completed two experimental trials, one with an exotendon attached to each foot, and the other was performed with barefoot. Each experimental trial consisted of bilateral hopping in place for 60 s, at 140 beats per minute (2.33 Hz), with the guidance of a digital metronome. This frequency was selected as it is close to human’s natural preferred hopping frequency^46^. A standing calibration trial was also collected with the participant standing quietly, in both the barefoot and exotendon conditions. When participants were fitted with the exotendon, they were given time to walk, jog and hop in the device, prior to testing to familiarize themselves with using the device. During this time, the investigators determined if the device needed to be adjusted to optimize the fit for comfort and slippage.

Participants performed the experimental tasks on a split-belt instrumented treadmill with the belts stationary and surface horizontal (AMTI, force-sensing tandem treadmill, Watertown, MA, USA) which recorded the ground reaction forces from each foot at 800 Hz. The forces were filtered with a 2nd order low-pass, zero-lag, butterworth filter at 60 Hz. Ground reaction force data were used to identify hopping events such as foot-contact and toe-off, as well as to compute an inverse dynamics analysis. Three-dimensional (3D) motion capture data were recorded at 200 Hz using an optoelectronic motion capture system (Qualisys, Gothenburg, Sweden). Reflective markers were placed bilaterally on the MTP (head of the first and fifth metatarsal), the ankle (medial and lateral malleoli), knee (medial and lateral epicondyle), and hip (greater trochanter). Four additional tracking markers were placed bilaterally on the shanks and thighs. For the pelvis, markers were placed bilaterally on the anterior and posterior iliac spine. The main focus of the study was the left leg, with symmetry between legs assumed due to the fact the exotendons were attached to both feet. Additional markers were placed on the left foot in order to construct a 3-segment foot model to estimate longitudinal arch and metatarsophalangeal (MTP) rotations, as described previously^47^. In order to track the toes as an additional segment, additional markers were placed on the most distal and dorsal heads of the 2nd and 4th proximal phalanges. In this model, the calcaneus, forefoot, and toes are modelled as individual rigid segments. The 3D marker trajectory data were filtered with a 2nd order low-pass, zero-lag, butterworth filter at 20 Hz.

### Joint kinematics & dynamics

Custom software developed in Matlab (Mathworks, Nattick MD, USA) was used to implement a sagittal plane inverse dynamics solution for the segments, joints, and plantar MTU of the left leg^6^. Markers were also placed on the right leg, pelvis, and trunk for the purposes of calculating centre-of-mass (COM) kinematics. All kinematic data were projected into the sagittal plane of the shank segment. Because markers on the calcaneus had to be moved to accommodate the placement of the exotendon, virtual markers were created that corresponded to the location of the original markers in the barefoot condition, within the coordinate frame of the calcaneus. This was accomplished by calculating the residual difference in position between each modified marker in the barefoot versus the exotendon static trials within the segmental frame of the calcaneus. During each motion trial, the positions of the virtual markers were calculated by adding this residual to the positions of the modified markers within the segmental frame of the calcaneus. For the exotendon conditions, the mass of the exotendon was added to the mass of the calcaneus.

Distally, ground reaction forces and moments were applied to the calcaneus, forefoot, and toe segments of the model based on the relationship between the position of the foot and the center of pressure as measured by the force plate, using a previously described method^6^. These forces and moments were transmitted from distal segments to proximal segments via the modelled rigid body chain to estimate the flexion–extension moment at each joint (MTP, arch, and ankle). Rotational powers of each joint were computed as the product of the moment and angular velocity of the joint. Joint work is calculated as the time integral of joint power. The quasi-stiffness of each joint was calculated as peak moment divided by peak angular deflection of the joint on for each full cycle of hopping (where a deflection of zero was defined as the joint angle during the static standing trial). Additionally the COM work rate was estimated as the dot product of the ground reaction force for each plate and the velocity of the COM (as estimated from the mass-weighted average of each segment’s COM velocity), as described previously^48^. COM work was estimated as the integral of COM work rate for each cycle of hopping.

### Plantar MTU kinematics & dynamics

**Figure 2:**
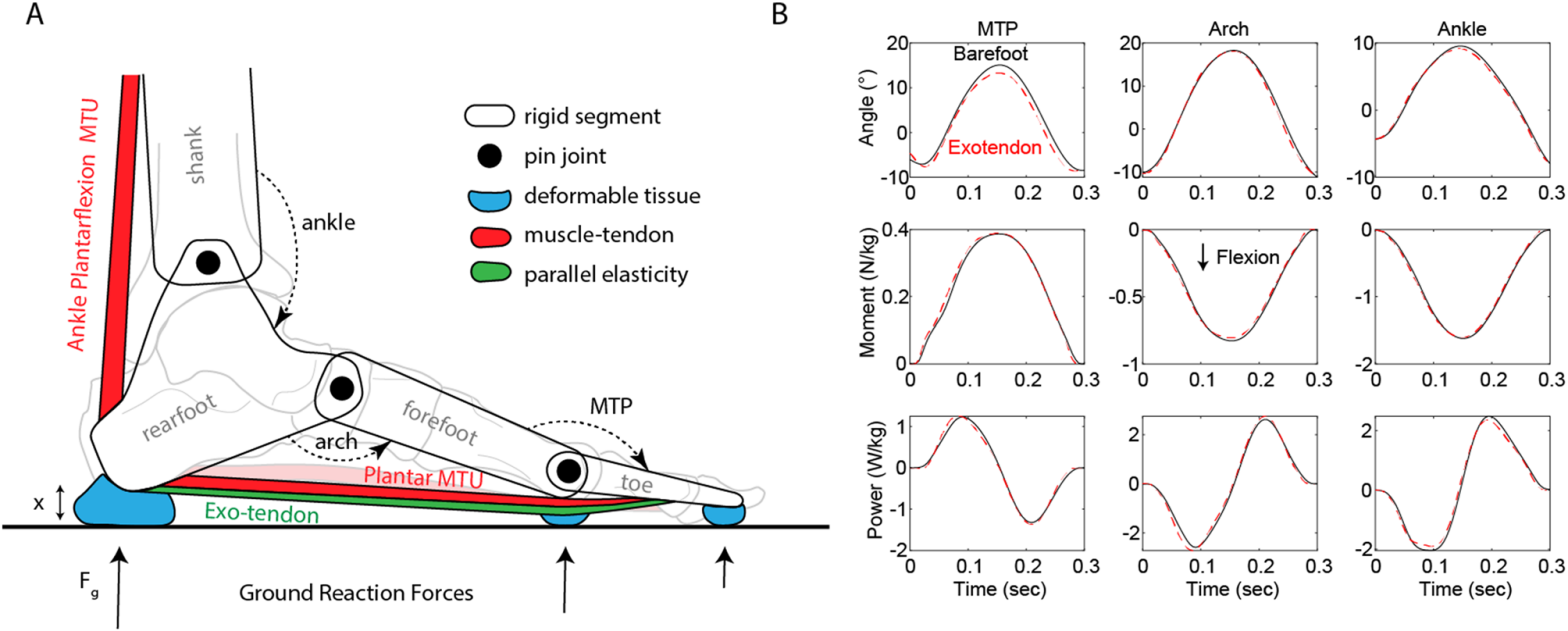
(**A**) A conceptual diagram of the inverse dynamics model used to take measurements of the foot and ankle during hopping. A series of four segments (shank, rearfoot, forefoot, and toe) are connected via 3 pin joints orientated perpendicular to the sagittal plane (ankle, arch, and MTP). The plantar MTU is a windlass mechanism acting across both the arch and MTP, through which both the passive elastic aponeurosis and intrinsic foot muscles generate force. During the experiment, an exotendon was also added and thereby included in measurements of the plantar MTU. Ground reaction forces as measured by a force plate are distributed across the three foot segments based on the relative positioning of each segment with respect to both the ground and the centre-of-pressure of the force plate (**B**) Angles, moments, and powers of the MTP, arch, and ankle for hopping at 2.33 Hz. Data are averaged across all participants, and the barefoot (black-line) is compared to wearing the exotendon (dashed red line).

The plantar MTU, the grouping of tissues and muscles acting via the windlass mechanism, was modelled as a muscle and tendon acting in parallel along the plantar aspect of the foot, spanning the arch and MTP (Fig. 2A). To quantify the dynamics of the plantar MTU and the exotendon, the length of the plantar MTU was estimated using a geometric model described previously^6,49^. A single estimate of MTU length was calculated, which incorporates motion data from the 1^st^, 2^nd^, and 4^th^ toes, as well as both medial and lateral aspects of the arch. The plantar MTU was modelled to originate from the inferior surface of the calcaneus, wrap about a circular surface at the MTP joint, and end with an insertion on the toe segment at half of that toe’s total length. The radius of the MTP joint was taken to be 7.45 mm as the average radius of all 5 metatarsal heads weighted by their contribution to total PF force, as estimated in a previous study^23^. The force produced by the plantar MTU was estimated by dividing the MTP moment by the lever arm between the MTP joint and the path of force that the MTU exerted onto the toe. The product of force and MTU velocity (which was calculated as the time derivative of MTU length) resulted in an estimate of MTU power. In conditions in which the exotendon was attached, we assumed that it acted in parallel along the same path as the plantar MTU path. Therefore, the moment and power estimated at the plantar MTU was taken to be the sum of the human and exotendon contributions. MTU stiffness was calculated analogously to joint quasi-stiffness, as the peak plantar MTU force divided by peak change in length of the plantar MTU on a per-cycle basis. Differences in the plantar MTU dynamics between barefoot and exotendon conditions were used to estimate the effects of the exotendon indirectly since we did not have access to separate measurements of the human foot and exotendon.

### Electromyography (EMG)

Activation of the abductor hallucis (AH), soleus (SOL), medial gastrocnemius (MG) and tibialis anterior (TA) were recorded from the left lower limb during each task using surface electromyography (EMG). The data was collected using Ag–AgCl electrodes with a 20 mm inter-electrode distance (Tyco Healthcare Group, Neustadt, Germany). EMG data was recorded at 1000 Hz (MA300, Motion Labs, LA, USA) and high-pass filtered at 5 Hz with a zero-lag butterworth filter. A root mean square (RMS) amplitude of the signal was calculated using a 25 ms moving window to produce an EMG RMS envelope for each muscle. The RMS envelopes were normalized to the mean of the highest 10 maximal RMS amplitudes across all hopping cycles for each participant. Both the peak level and the time-integrated activation level of this processed signal were used to estimate the magnitude of muscle activation within the muscle over the stance phase of a given hopping cycle. The peak activation was used primarily to indicate how the exotendon affected the muscles activation associated with peak force generation compared to barefooted hopping. The search window for the peak activation of AH, MG, and SOL was constrained to the first 50% of stance phase to ensure that the first peak was consistently selected (Fig. 4). In contrast, the peak activation of TA was defined as the maximum level of activation just prior to losing contact with the ground (Fig. 4).

### Statistics

All measures of muscle activation and gait mechanics were analysed with a linear mixed-effects model. The main independent variable of interest was whether the exotendon was worn during hopping and was treated as a fixed-effect in the model. Participants were treated as a random effect such that each participant had their own intercept when fitting the model for each measure, such that differences in average levels of a measure across participants did not influence the analysis of the exotendon. For each measure, the model in Wilkinson notation can be written as:

$$Measure=Exotendon+(1|Participant)$$

For the stiffness of joints and the plantar MTU, muscle activation was also included as a fixed effect as an attempt to separate changes in stiffness due to the exoskeleton as opposed to muscles changing their activation levels. This model can be written as:

$$\mathrm{Stiffness}=\mathrm{Exotendon}+\mathrm{Muscle \,Activation}+(1|\mathrm{Participant})$$

For the joints of the foot and plantar MTU, the muscle activation was taken to be the integrated AH activation during contact with the ground whereas for the ankle joint, the muscle activation was taken to be the integrated activation of SOL (Table 3). The exotendon was considered to have a significant effect when the p-value for the *F-*test on the Exotendon fixed-effect coefficient had a p-value of less than 0.05.

## Results

### Full body kinematics & COM work

Across all 9 participants, a total of 2475 full cycles (touch-down to subsequent touch-down) of hopping were analyzed across both conditions, with an average of 137.5 hopping cycles per participant per condition recorded. We verified that there was no significant difference between hopping frequency in barefoot and exotendon conditions, 2.33 ± 0.0016 Hz and 2.33 ± 0.0022 Hz respectively (p = 0.41). Participants spent slightly less time on the ground as a percentage of a whole hopping cycle while wearing the exotendon, at 68.4 ± 3.6% and 67.3 ± 3.7% (for barefoot and exotendon respectively, p < 0.01). At the COM, the amount of negative work (− 0.451 J/kg barefoot, versus -0.438 J/kg exotendon, p = 0.49) and positive work (0.449 J/kg barefoot, versus 0.442 J/kg exotendon, p = 0.73, Table 1) during contact was not significantly different between conditions.

**Table 1:** Measures presented are averaged across all participants and are presented in the format mean ± standard deviation for the barefoot and exotendon conditions.

Measure	Barefoot	Exotendon	Units	p
Peak MTP excursion	25.5 ± 3.4	23.9 ± 3.5	°	< 0.001
Peak arch excursion	30.4 ± 4.6	29.8 ± 3.5	°	< 0.001
Peak ankle excursion	18.0 ± 3.8	17.1 ± 3.2	°	< 0.001
Peak plantar stretch	0.0151 ± 0.0046	0.0154 ± 0.0039	m	0.003
Mean MTP moment	0.217 ± 0.028	0.225 ± 0.031	Nm/kg	< 0.001
Mean arch moment	0.426 ± 0.079	0.429 ± 0.067	Nm/kg	0.091
Mean ankle moment	0.806 ± 0.12	0.817 ± 0.12	Nm/kg	< 0.001
Mean plantar force	29.1 ± 3.8	30.2 ± 4.1	N/kg	< 0.001
MTP stiffness	0.0195 ± 0.0049	0.0224 ± 0.0074	Nm/°/kg	< 0.001
Arch stiffness	0.0306 ± 0.0073	0.0303 ± 0.0069	Nm/°/kg	0.18
Ankle stiffness	0.108 ± 0.038	0.105 ± 0.24	Nm/°/kg	0.63
Plantar stiffness	4240 ± 3100	4620 ± 5520	N/m/kg	0.037

The 5th column has the p-value for the fixed efficient coefficient for the exotendon condition in the linear mixed effects model, where p < 0.05 was considered significant.

### Joint kinematics & dynamics

**Figure 3:**
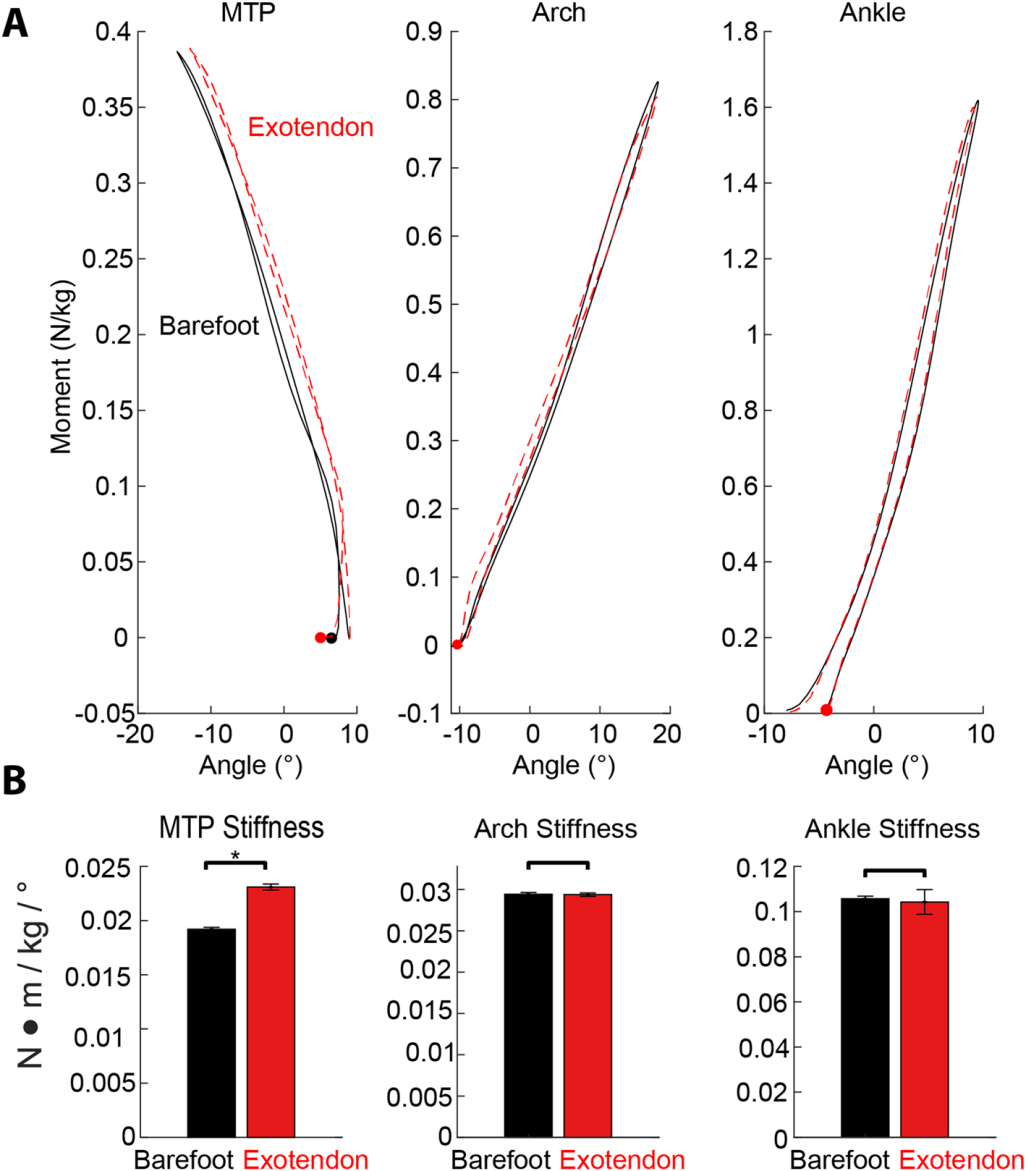
(**A**) Sagittal moment vs angle for the MTP, Arch, and Ankle, showing an approximately linear relationship with little hysteresis. Compare barefoot (black solid line) to exotendon (dashed red line) averaged across participants. The exotendon stiffens the MTP throughout the hopping cycle, whereas the estimated linear stiffness of the arch and ankle were largely unchanged. Dots represent first contact with the ground. (**B**) A linear fit between peak joint moment and angular deflection was used to estimate a net stiffness for the joint. The bar plot shows the mean ± S.E. for this stiffness value across the two conditions. * denotes significance, as tested by the linear mixed model which also takes statistical account for differences in stiffness that could have resulted purely from changes in muscle activation of AH for the MTP and arch, or from SOL for the ankle.

Of the joints measured, the exotendon most influenced the dynamics of the MTP. The MTP underwent a smaller range of motion and exhibited a slightly increased moment when the exotendon was worn (Fig. 2B). Peak angular excursion of the MTP joint was reduced from 25.5° to 23.9° (p < 0.001) while wearing the exotendon, whereas the mean moment was increased from 0.217 Nm/kg to 0.225 Nm/kg (p < 0.001, Table 1). Estimating a linear stiffness from the moment–angle hysteresis plots (Fig. 3) showed that the MTP joint increased in stiffness from 0.0195 Nm/°/kg in bare feet to 0.0220 Nm/°/kg wearing the exotendon (p < 0.001, Table 1). The range of motion and mean moments of the ankle and arch followed similar patterns of changes between conditions, although there was no significant difference in stiffness at these joints (p = 0.18 and p = 0.63 for the arch and ankle respectively, Table 1). The arch was stiffened during the initial loading phase of hopping (Fig. 3A) without significantly affecting peak deflection or moment and therefore the overall measurement of quasi-stiffness for the arch (Fig. 3).

### Joint work

The amount of negative and positive work done at the MTP was not significantly different across conditions (p = 0.065 and p = 0.63 respectively, Table 2). The amount of negative work at the arch increased from − 0.203 J/kg to − 0.211 J/kg (p < 0.001) whereas negative work at the ankle decreased from − 0.177 J/kg to − 0.166 J/kg (p < 0.001, Table 2). Similarly, the positive work at the arch increased from 0.190 J/kg to 0.193 J/kg (p = 0.004), whereas the positive work at the ankle decreased from 0.212 J/kg to 0.200 J/kg (p < 0.001, Table 2).

**Table 2:** Measures presented are averaged across all participants and are presented in the format mean ± standard deviation for the barefoot and exotendon conditions.

Negative work (J/kg)			Source	Positive work (J/kg)		
Barefoot	Exotendon	p		Barefoot	Exotendon	P
− 0.0971 ± 0.022	− 0.098 ± 0.022	0.065	**MTP**	0.0956 ± 0.026	0.0960 ± 0.024	0.63
− 0.203 ± 0.053	− 0.211 ± 0.050	< 0.001	**Arch**	0.190 ± 0.036	0.193 ± 0.039	0.004
− 0.177 ± 0.065	− 0.166 ± 0.058	< 0.001	**Ankle**	0.212 ± 0.080	0.200 ± 0.074	< 0.001
− 0.372 ± 0.16	− 0.415 ± 0.13	< 0.001	**Plantar MTU**	0.329 ± 0.13	0.360 ± 0.12	< 0.001
− 0.451 ± 0.43	− 0.438 ± 0.47	0.49	**COM**	0.449 ± 0.44	0.442 ± 0.43	0.73

The 3rd column has the p-value for the fixed efficient coefficient for the exotendon condition in the linear mixed effects model, where p < 0.05 was considered significant.

### EMG activation

**Figure 4:**
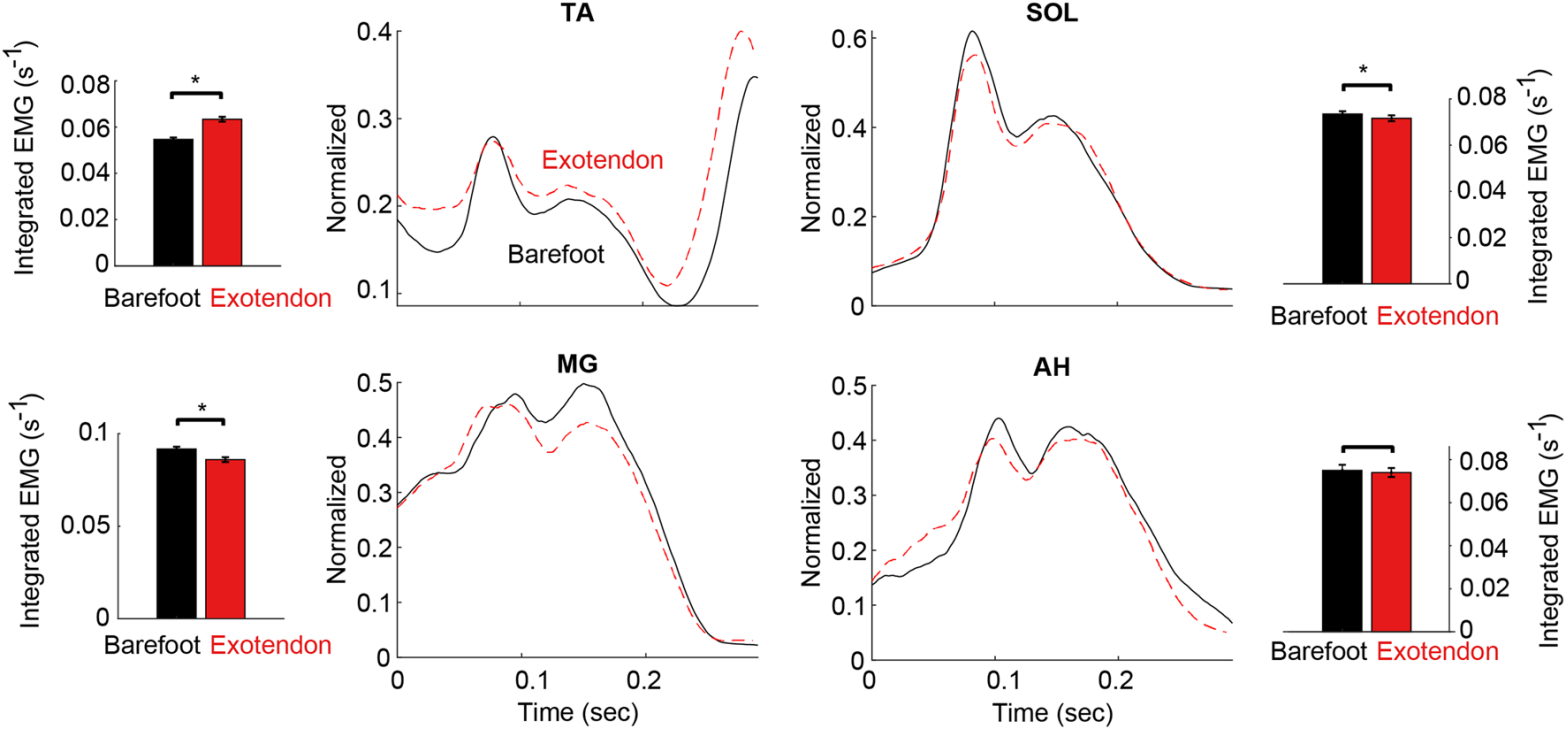
Electromyography data of four foot–ankle muscles during stance phase of hopping at 140 Hz averaged across all participants. Data are rectified, bandpass filtered, filtered with a moving RMS window, and normalized to the average of the top 10 peak activations of each muscle while running at 3 m/s on a per-participant basis. The continuous time plots show the activation patterns of each muscle during the contact phase of a hopping cycle, comparing barefoot (black line) to the exotendon (dashed red). The exotendon tended to decrease peak activation of the foot muscle AH and ankle plantarflexors (MG and SOL), whereas it tended to increase the mean activation of the ankle dorsiflexor TA across the entire cycle. Additionally, integrated activation levels are shown as bar plots adjacent to each of the time series plots, with an asterisk denoting significance between barefoot and exotendon data as estimated by the linear mixed effects model.

The time-series data of muscle activation exhibited similar patterns comparing barefoot to exoskeleton conditions but with significant differences in peak and integrated activation levels (Fig. 4). Compared to barefoot, wearing the exotendon decreased peak activation of the ankle plantar flexors SOL and MG by 4.7% and 3.6% respectively (both p < 0.01, Table 3). Peak activation of AH remained relatively constant (− 1.8%, p = 0.31). In contrast, peak activation of TA increased by 17.8% with the exotendon applied (p < 0.01). These patterns were similar for the time-integrated EMG activation (Table 3).

**Table 3:** Peak and integrated EMG measures presented are averaged across all participants and are presented in the format mean ± standard deviation for the barefoot and exotendon conditions.

Measure	Barefoot	Exotendon	Units	p
Max AH	0.690 ± 0.61	0.677 ± 0.49	None	0.31
Max SOL	0.765 ± 0.37	0.729 ± 0.37	None	< 0.001
Max MG	0.697 ± 0.21	0.672 ± 0.22	None	< 0.001
Max TA	0.507 ± 0.27	0.597 ± 0.31	None	< 0.001
Integrated AH	0.0752 ± 0.075	0.0739 ± 0.060	s^−1^	0.4
Integrated SOL	0.0734 ± 0.038	0.0716 ± 0.041	s^−1^	0.0016
Integrated MG	0.0916 ± 0.031	0.0859 ± 0.033	s^−1^	< 0.001
Integrated TA	0.0547 ± 0.029	0.0634 ± 0.034	s^−1^	< 0.001

The 5th column has the p-value for the fixed efficient coefficient for the exotendon condition in the linear mixed effects model, where p < 0.05 was considered significant.

### Plantar MTU kinematics, dynamics, & work

**Figure 5:**
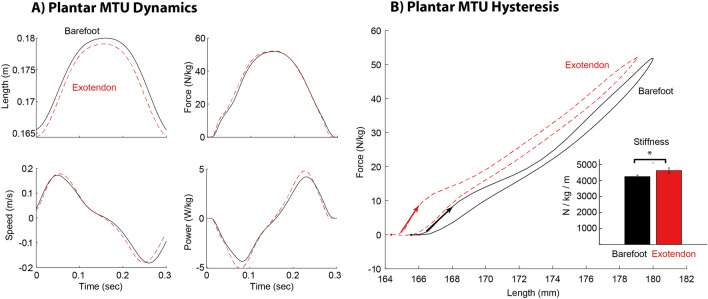
(**A**) Modelled dynamics of the plantar MTU, averaged across participants and compared between barefoot (solid black line) and exotendon (dashed red-line) (**B**) Force–displacement graph of the plantar MTU with arrows are located at initial contact for the hopping cycle and denote the stiffness at that time. Inset bar plot shows the difference in the estimated linear stiffness of the MTU between barefoot and exotendon conditions.

The exotendon targeted the dynamics of the plantar MTU where perhaps the clearest differences between barefoot and exotendon were apparent. With participants wearing the exotendon, the externally assisted plantar MTU reached a shorter peak length, exerted a higher force, and generated more negative and positive power (Fig. 5A). The linear stiffness (while accounting for differences in activation of AH) of the plantar MTU significantly increased (Fig. 5B, Table 1), changing from 4240 N/m/kg barefoot to 4620 N/m/kg when externally assisted with the exotendon (p = 0.037), although the estimated linear stiffness was quite variable as noted by the large standard deviations in the measures (Table 1). This increase in plantar MTU stiffness is equivalent to a 9.0% change from barefoot to exotendon. Finally, although the peak length of the plantar MTU was shortened, the excursion from its shortest to longest length was greater compared to barefoot, but by less than 0.5 mm (p = 0.003, Table 1).

Both negative and positive work of the plantar MTU increased when wearing the exotendon, by about 11.6% and 9.4% respectively (p < 0.001, Table 2). The smaller amount of additional positive work returned at the plantar MTU (compared to the larger increase in negative work) indicates that the exotendon performed work at about a 72% efficiency.

## Discussion

We developed and implemented a biomimetic exotendon to act in parallel with the plantar aponeurosis and intrinsic foot muscles. As hypothesized, the exotendon increased the stiffness of the plantar MTU, by approximately 9%, during a bilateral hopping task. This stiffening of the foot increased the amount of elastic work performed along the plantar MTU pathway by 9.4% without significant changes to the activation of AH, which based on our previous studies, likely represents other intrinsic foot muscles^49,50^. These effects corresponded with a decrease in peak activation of MG and SOL (4.7% and 3.6% respectively). Positive ankle work also reduced (by 5.5%) but dynamics of the COM remained unchanged. Since ankle plantarflexion is responsible for a significant amount of the total cost in locomotion and hopping^39,48,51,52^, the reduction of both ankle work and plantarflexion muscle activity suggests the exotendon may be able to reduce the cost of ankle plantarflexion for hopping. The direct effect of the device on metabolic cost needs to be tested, since a reduction in muscle activation and joint work does not always result in a reduction in muscle work or metabolic cost^39^.

While the effects of footwear on muscle activation and joint work have not extensively been studied during hopping, shoes with carbon-fiber plates can reduce the cost of running by about 4% via a stiffening of the foot^9^. Although our results are not directly applicable to running, the vertical bouncing motion of the COM is similar for both activities^40^. As such, we expect that our results would be applicable to running to a certain extent. Based on our results, the mechanism by which such performance enhancement occurs during bouncing motions is the increased stiffness and power output of the windlass mechanism, which in turn reduces ankle work. Mechanically, this could operate through the coupling of the windlass mechanism and the ankle plantar flexors in series about the ankle. Since the average activity of AH was unchanged, the results support the hypothesis that footwear can increase performance by increasing the efficiency of ankle movements as opposed to directly decreasing the active muscle work in the intrinsic foot muscles.

We also observed a significant increase in the activation of TA throughout contact with the ground, which is likely a result of the tension of the device pulling the foot and toes downwards. Increased activation of TA may be required to help appropriately place the toes down as the body is landing, and pick them up as the body is pushing off while the device is worn. The increased mass of the foot due to the device may also play a role in increasing activation; however, as noted previously the mass of the device is quite small and therefore not expected to result in such a large change in activation. Regardless of the exact reasons, it is currently unclear to what extent the cost of increased TA activation may offset reductions in cost from reduced plantarflexion work. Nevertheless, our findings are consistent with exoskeletons that stiffen the ankle, which tend to reduce MG and SOL activity at the expense of increased TA activity^34,53,54^.

While the exotendon passively increased both the stiffness and work output of the plantar MTU, the MTP and foot arch exhibited more intricate changes that warrant discussion. Although these two joints together behaved similar to a linear spring (Fig. 5B), the increase in stiffness due to the exotendon occurred at the MTP (with no changes in work), whereas the increased work output of the foot occurred primarily at the foot arch (with no changes in stiffness). The reason that the additional elastic work appears at the arch and not the MTP may be due to how the MTP joint is pinned to the ground for most of the time the foot is in contact, such that releases in tension must occur through the arch which is free to move (the calcaneus does not generally contact the ground during hopping). The fact that in normal running the amount of positive work returned at the MTP is much smaller than the negative work is indicative of this idea^21,22^.

Although the estimates of elastic work increased, as measured at the plantar MTU and the foot joints (MTP + arch), the magnitude of this increase was not the same across the two measurements. Positive work at the plantar MTU increased by about 0.03 J/kg whereas the summed MTP and arch joint positive work only increased by about 0.003 J/kg (Table 2). This difference in magnitude could potentially be explained by the differing set of assumptions required for each measure which may skew the results in opposite ways. Firstly, the joint methodology assumes that the foot is entirely rigid between each of the respective joints, an assumption which is clearly false (e.g. feet lengthen under load) and would tend to underestimate changes to mechanical work within the foot. In contrast, the plantar MTU methodology presented here assumes that all of the moment at the MTP is generated via the plantar MTU, which may overestimate its force since uni-articular muscles and other passive structures may contribute to this moment, tending to overestimate the energy moving through the plantar MTU. As a result, these measurements could be considered upper and lower bounds on the amount of elastic work in the foot, with the actual changes in energy somewhere in between these bounds. Nevertheless, the trend of increased elastic work and stiffness is consistent across both methodologies and in that sense, both support the same overall interpretation of the effects of the exotendon on the mechanics of the foot.

More detailed experiments are required to determine the exact amounts of energy going through the foot across both the plantar MTU, uni-articular muscles, and passive structures of the foot. In this regard, the methodology used to distribute force across the segments of the foot can greatly affect these estimates, especially at the MTP. The common methodology of assuming zero moment at the MTP until the centre-of-pressure (COP) crosses the joint axis has been shown to overestimate the peak MTP moment and power (COP-progression method^55^). The methodology used in this paper to assign force to the segments of the foot^6^ addresses these issues (without the use of additional sensors) using a probabilistic model consistent with the progression of the COP and it’s relation to the motion of the segments of the foot as measured by motion capture. This methodology estimates a lower value for peak MTP moment and power than the COP-progression method. For example, we reported that the mean moment measured at the MTP during stance phase for the barefoot condition was 0.217 Nm/kg (Table 1) whereas the COP-progression methodology would have resulted in an estimate of 0.249 N/kg. Although these lower estimates of lower MTP moment and power are consistent with the conclusions from experiments using multiple force plates and pressure insoles^55^, the methodology has yet to be validated in a controlled study.

Perhaps the most salient issue with wearable assistive devices is that adding mass to the body leads to less efficient movements^43–45^. This is particularly true of devices attached to the distal lower limb, where the added mass greatly influences limb moment of inertia. Devices which have heavy elements such as rigid exoskeletons or powerful motors can struggle to provide performance benefits^56^. As such, there is an increased focus to use soft materials and ergonomic designs that provide benefits in order to alleviate this problem^57–59^. One potential advantage of the exotendon over normal footwear is that it operates via tension, as opposed to shoes with carbon fiber plates which stiffen the foot by bending. The exotendon design mimics the structure of muscles and tendons in the body which generate force through tension and enables the device to be very light with a total mass of only 55 g. In comparison, a shoe with a carbon fiber plate in a previous study was about four times heavier than the exotendon with a mass of about 240 g, although this mass does include the cushioning elements of the shoe which are important for other reasons than performance^8,21^. Additionally, since the exotendon contours to the shape of the foot directly, it can be worn within a shoe, which if worn in conjunction with a foot-stiffening shoe could increase foot stiffness beyond what either device could accomplish independently.

The ability of the exotendon to stiffen the foot is dependent on the effectiveness of attaching the device at the toes and heel. Any slack at these attachment points greatly decreases the ability of the device to effectively transfer energy across the foot. In one experiment on running with an exoskeleton, it was estimated that slippage of the exoskeleton accounted for a 50% decrease in energy transfer between the human and the device^60,61^. While we made our best effort in ensuring that slipping was minimal, it certainly would have still reduced the effectiveness of the device. However, because the dynamics of the body were measured directly, we can be sure that the measurements reported here are the total effect of the device (including its intended elastic motion and any unwanted slipping, friction, and slack) on the participant. We found that the 74% of the additional work absorbed by the assisted plantar MTU was returned as positive work during push off, a value which can presumably be increased by optimizing the attachment to the body. Finally, because we tested only a single exotendon, we are unable to explain the relationship between mechanical properties of the exotendon (such as stiffness and rest length) and outcome measures such as ankle work and muscle activity. The fact that the exotendon shifted the force–displacement curve towards shorter plantar MTU lengths (Fig. 5B) suggest that the rest length of the exotendon may be an important parameter for the function of the device. Further study is needed to understand how to best design exoskeletons with mechanical properties that optimize measures of performance.

The highly elastic nature of cyclic hopping is particularly well suited for testing the potential benefits of wearing a plantar exotendon. The foot and ankle joints operated nearly elastically as observed in the work loops (Fig. 3A), suggesting that the dynamics of linear elastic elements added in parallel with the muscles and tendons match the required dynamics of the experimental task. On the other hand, the work loops of the body’s joints in running are more nonlinear^25,41^. It is likely that such differences are the result of increased energy being dissipated from impact with the ground^2,62^ and increased active muscle work in order to compensate for this loss in energy^5^. Passive elastic devices have negligible ability to modulate the net energy of a system and therefore would be expected to have little effect in reducing the metabolic costs associated with these energetic requirements.

The device may increase the cost of swinging the leg in running, since we observed increased activation of TA, although we expect that the device may still be beneficial to performance since both exoskeletons on the ankle^34^ and shoes^9^ have successfully increased economy in running in spite of similar potential drawbacks. The evaluation of an assistive device seeking to improve the performance of a task should take into account the degree to which the targeted pathway of the device contributes to the total cost of the task. In this regard, the relatively small but significant increase in plantar MTU stiffness of 9% may only be directly applicable for competitive athletes in which small gains of performance may be important, and in which the vertical bouncing dynamics of hopping are expected to play a major role. However, such a device also has the potential to inform the design of orthotics, prostheses, and robotic appendages which impact a broader range of people. Prostheses which emulate the loading response of the ankle can reduce the metabolic cost of unilateral transtibial amputees during walking ^63^, for example.

This study provides evidence in support of the concept that lightweight elastic footwear mimicking the anatomy of the plantar MTU can lower muscle activation and mechanical work requirements in the ankle plantarflexors, at least acutely during hopping. While it is still unclear to what extent these devices can meaningfully improve performance economy across the range of activities a person may undertake during a day (running, walking, standing, etc.), our results nevertheless provide insight into the anatomical pathways through which previous foot-stiffening shoes may have provided performance benefits. Analyzing the function of the foot by measuring the dynamics of the plantar MTU as opposed to focusing on individual joints may be better suited for evaluating the mostly elastic function of the foot in hopping. As such, it may be beneficial to focus on stiffening the foot with devices that mimic the structure of the plantar MTU, which could be placed within normal footwear that may have carbon plates embedded if additional stiffening is required. Such devices appear to provide benefits to the wearer by reducing muscle activity for ankle plantarflexion as opposed to reducing activation within the foot or minimizing energy dissipation.

## Data Availability

The dataset used in this work is available online (10.6084/m9.figshare.14044253).
